# The ‘Nature Impact’ feasibility study protocol: a mixed methods feasibility study of a group- and nature-based health intervention for adults experiencing mild to moderate anxiety, depression and/or stress

**DOI:** 10.1186/s40814-026-01875-x

**Published:** 2026-06-22

**Authors:** Signe Salanson Jensen-Smidt, Louise Sofia Madsen, Lisa Gregersen Oestergaard, Dorthe Varning Poulsen, Knud Ryom, Nanna Holt Jessen

**Affiliations:** 1 Research Unit for General Practice, Aarhus, Denmark; 2 DEFACTUM, Aarhus, Central Denmark Region Denmark; 3 Department of Geosciences and Natural Resource Management, University of Copenhagen, Frederiksberg, Denmark; 4 Department of Public Health, University of Aarhus, Aarhus, Denmark

**Keywords:** Nature-based health interventions, Mental health, Feasibility study, Mixed methods, Protocol, Complex interventions

## Abstract

**Background:**

Anxiety, depression and stress-related conditions are prevalent and growing public health challenges, impacting individuals’ quality of life and placing significant strain on healthcare systems globally. These complex conditions rarely respond to a single mode of treatment, underscoring the importance of diverse and complementary approaches. Nature-based health interventions (NBHIs) represent one such perspective, offering novel opportunities to address mental health by engaging with natural environments. While international interest in NBHIs is growing, evidence on how these interventions can be systematically developed and implemented remains limited. The aim of this study protocol is to report the planned Nature Impact Feasibility Study.

**Methods:**

The Nature Impact Feasibility Study is a one-armed, mixed methods feasibility study aiming to evaluate a complex, group-based NBHI developed using the Medical Research Council framework for complex interventions. The intervention will be conducted across three sites in Denmark: Glostrup outpatient clinic, Kolding and Silkeborg municipality centres. The intervention consists of group-based NBHI programmes delivered weekly over 10 consecutive weeks, facilitated by experienced health professionals. The feasibility evaluation will examine three core domains. These include (1) the implementation process, including fidelity, dose, reach, adaptations and acceptability; (2) mechanisms of change and (3) contextual factors influencing implementation and intervention processes. Data will be collected through participant observations, focus group interviews, registration forms and participant questionnaires assessing mental health outcomes and the participants’ relationship to nature. Data will be analysed using a mixed methods convergent design. Quantitative and qualitative data will be analysed separately and then integrated thematically during interpretation.

**Discussion:**

By reporting the study protocol for the Nature Impact Feasibility Study in advance, the project enhances transparency, allows for critical appraisal of the planned design and methods and strengthens the foundation for the subsequent feasibility evaluation and future large-scale trials. The Nature Impact Feasibility Study will provide valuable insight into delivering NBHIs for adults experiencing mild to moderate anxiety, depression and/or stress, while informing intervention refinement, outcome measures and future research aimed at investigating the potential of integrating NBHIs into existing practices for treating anxiety, depression and stress.

**Trial registration:**

NCT06981000. Registered on March 13, 2025.

**Supplementary Information:**

The online version contains supplementary material available at https://doi.org/10.1186/s40814-026-01875-x.

## Background

Mental health problems constitute a world-wide growing public health concern, with significant implications for individuals, communities and the healthcare system [1]. According to the Global Burden of Disease Study 2021, the global age-standardised incidence rate (ASIR) of mental disorders reached 5456 per 100,000 individuals, representing a 15% increase since 1990. Anxiety and depression are the most prevalent contributors to this increase [1]. Despite this growing burden, mental health remains severely underfunded. Mental health receives, on average, only 2.1% of the national health budgets [2]. This underfunding limits the ability of healthcare systems to meet the growing and increasingly complex demands, particularly among patients who do not respond to standard treatments.

While clinical treatment and pharmacotherapy are essential components of mental healthcare, growing evidence indicates that these approaches can be insufficient. This is due not only to financial constraints, but also to the complex needs of patients who experience treatment resistance to standard pharmacological treatment [3, 4]. For example, a major review found that approximately one-third of individuals with depression do not respond adequately to standard pharmacological treatment [4]. This situation underscores the need for alternative and complementary approaches that can promote mental well-being and reduce the wider burden on healthcare systems.

One such approach gaining international attention is nature-based health interventions (NBHIs), which involve structured therapeutic engagement with natural environments. A growing body of research indicates that NBHIs can alleviate symptoms of anxiety, depression and stress [5–8]. However, despite international interest, there is a demand for a comprehensive, evidence-informed framework for the systematic development, implementation and evaluation of NBHIs in clinical and municipal healthcare settings [5–8]. To address this gap, a complex intervention development process is undertaken in the Nature Impact Feasibility Study, guided by the UK Medical Research Council’s (MRC) framework for developing and evaluating complex interventions [9]. This framework emphasises the importance of understanding not only the intervention and its outcomes but also the broader contextual factors that may influence implementation. Contextual factors refer to a broad range of conditions, e.g., organisational structures, participants’ mental health status, physical settings and weather conditions.

The present study protocol aims to report the planned Nature Impact Feasibility Study.

## The Nature Impact Feasibility Study

### Aim of the Nature Impact Feasibility Study

The primary aim of the Nature Impact Feasibility Study reported in this protocol is to evaluate the feasibility of the intervention. This will be done by examining three core domains: (1) the implementation process, including fidelity, dose, reach, adaptations and acceptability; (2) the mechanisms of change; and (3) contextual factors and how these influence the implementation and the mechanisms of change. The findings from this evaluation will provide a foundation for refining a programme theory and an intervention manual and guide the planning of future evaluation and effect studies.

The secondary aim of the Nature Impact Feasibility Study is to explore preliminary changes in participants’ mental health status during the intervention period, including mental well-being, symptoms of anxiety, depression and stress, life satisfaction and their connection to nature. This assessment aims to evaluate the suitability of the selected outcome measures and to provide insight into the intervention’s potential efficacy.

Building on the MRC framework [9], the Nature Impact Feasibility Study follows a structured approach consisting of two key phases: an initial intervention development phase and a subsequent feasibility testing phase, the latter of which is reported in detail in this protocol.

### Intervention development phase

The intervention development phase included several components. These comprised a systematic literature review on effects of NBHIs for individuals experiencing symptoms of mild to moderate anxiety, depression and/or stress [8], a narrative review of the mechanisms of change for NBHIs, stakeholder dialogue meetings, a Delphi survey with experts on group- and nature-based interventions [10]. In addition, a programme theory [11] and an intervention manual were developed to guide practitioners with NBHIs for future group- and nature-based interventions. The programme theory, presented through the logic model, outlines the relationships between the intervention’s inputs, core elements, mechanisms of change and anticipated outcomes. The model is intended as a flexible, practice-oriented tool to guide health professionals in the planning, delivery and evaluation of NBHIs. By allowing for contextual adaptation, the model has supported the design of interventions that are both evidence-informed and contextually appropriate.

The programme theory is informed by the Delphi process, which identified the following core mechanisms of change in NBHIs: nature interaction, social community and physical activity, alongside related processes such as sensory experiences, body awareness and self-reflection. These mechanisms have informed the design of the present intervention and are operationalised through group-based activities in natural environments, such as guided nature exposure, shared reflections and light physical group-activities, which are expected to support mental health through these interrelated processes. This is grounded in a bio-psycho-social approach.

Preparatory activities to support consistent delivery included workshops on co-creation and contextual adaptations, site visits, development of the intervention manual and competence development sessions across the three study sites (November 2024–March 2025).

Health professionals responsible for delivering the intervention have tailored the activities to suit the participants’ needs and resources using the potential of a specific natural environment. Potential risks have been assessed and minimised through the selection of suitable natural environments, appropriate equipment and thorough instructions.

Health professionals have been encouraged to create a safe, inclusive and supportive environment where participants can feel comfortable, both physically and psychologically. Accessibility and individual needs have been considered, to ensure that all participants can safely engage in the activities.

**Fig. 1:**
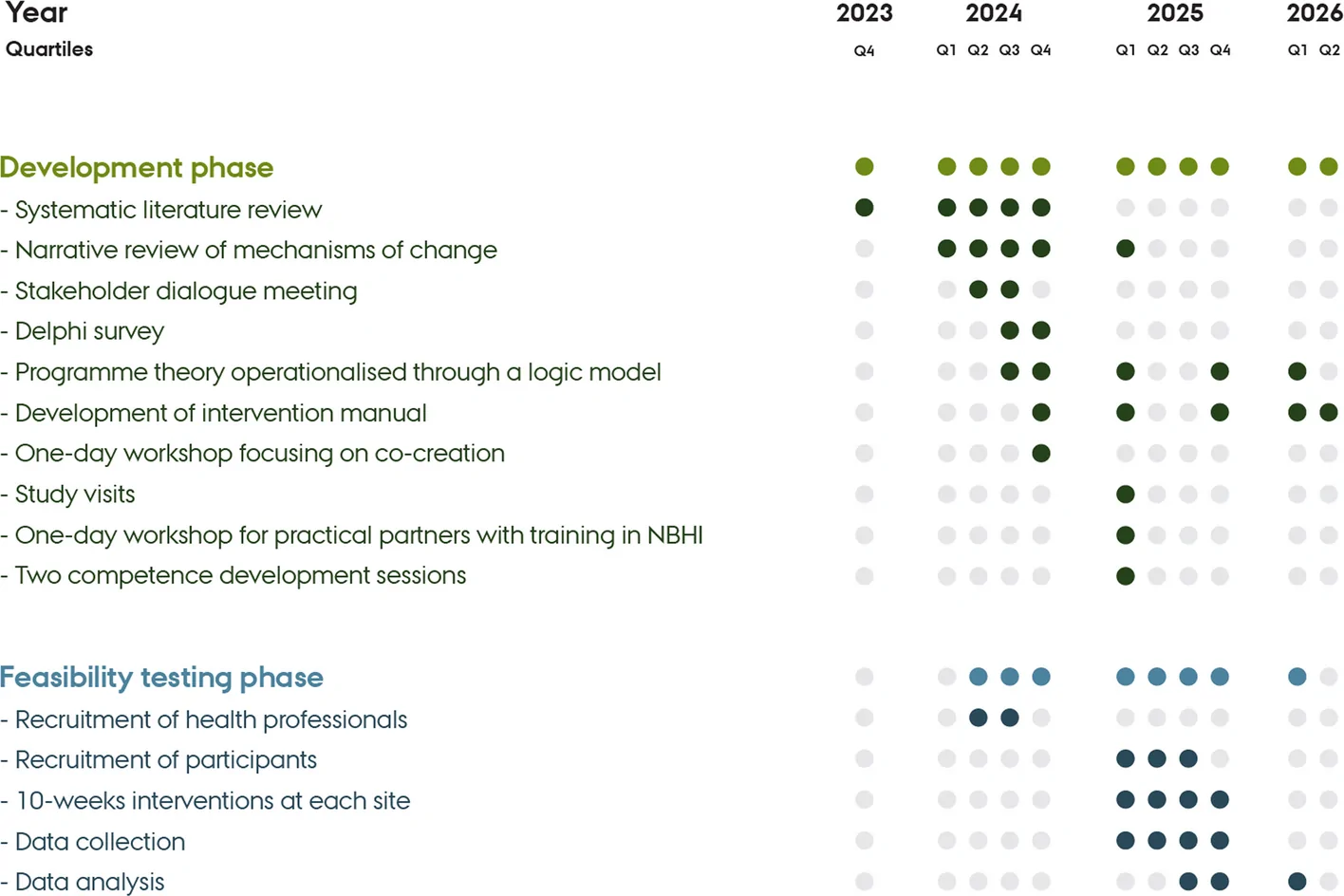
Gantt chart displaying the timeline of the development and feasibility testing phases

Figure 1 presents a detailed timeline of the intervention development and feasibility testing phases, encompassing all major project components. The indicated time frames for the articles refer to data collection only and does not include the writing process.

## Methods

### Research design for the Nature Impact Feasibility Study

The feasibility study is designed with a one-armed, mixed methods, prospective design. As defined by Thabane and Lancaster, a feasibility study can inform the design of future main trials by assessing the feasibility and by identifying potential challenges [12]. A mixed methods convergent design [13] is used to pragmatically explore the feasibility parameters through qualitative and quantitative data, which are analysed separately and integrated thematically during interpretation. The design integrates quantitative and qualitative data to provide a comprehensive understanding of the feasibility and implementation in real-world settings. The domains, research questions and corresponding data collection methods are summarised in Table 1.

**Table 1:** Table of domains, research questions and data collection methods

Domain	Research questions	Data collection
Implementation (dose)	What proportion of the scheduled sessions do participants attend?	-Post intervention participant questionnaires
Implementation (reach)	Who are the participants?	-Participant questionnaires -Participant registration forms -Focus group interviews with the participants -Participant observation
	Are the partners able to identify the right participants and does the intervention reach the intended target group?	-Focus group interviews with health professionals -Participant registration forms -Participant questionnaires -Participant observation
	Have any of the participants failed to complete the intervention (dropout) and what factors have contributed to their withdrawal?	-Focus group interviews with health professionals -Participant questionnaires
Implementation (fidelity)	Is the intervention delivered as planned, and how are its components put into practice?	-Participant observation -Focus group interviews with health professionals -Intervention registration form
Implementation (adaptation)	Which adaptations do the health professionals make to the content of the intervention and why?	-Intervention registration form -Focus group interviews with health professionals
	How does the local adjustments adhere to the programme theory?	-Participant observation -Focus group interviews with health professionals
Implementation (acceptability)	To what extent does the intervention match the participants’ functional ability and mentally condition?	-Participant observations -Focus group interviews with participants
	Are the purpose and content of the intervention clear to the participants and the health professionals?	-Participant observations -Focus group interviews with participants -Focus group interviews with health professionals
	To what extent do participants perceive the intervention as impactful?	-Focus group interviews with participants
	Are the professionals’ competencies aligned with the requirements of the intervention?	-Participant observations -Focus group interviews with health professionals
Mechanisms of change	Does the intervention function as intended and are the anticipated mechanisms of change activated as contributors to the observed outcomes?	-Participant observations -Intervention registration forms -Focus group interviews with health professionals -Focus group interviews with participants
Contextual factors	How do contextual factors influence the implementation of the intervention?	-Participant observations -Focus group interviews with health professionals -Focus group interviews with participants
	How do contextual factors influence the mechanisms of change?	-Participant observations -Focus group interviews with health professionals -Focus group interviews with participants
Mental health status	Have the participants experienced any changes in their mental health during the intervention?	-Participant questionnaires

**Fig. 2:**
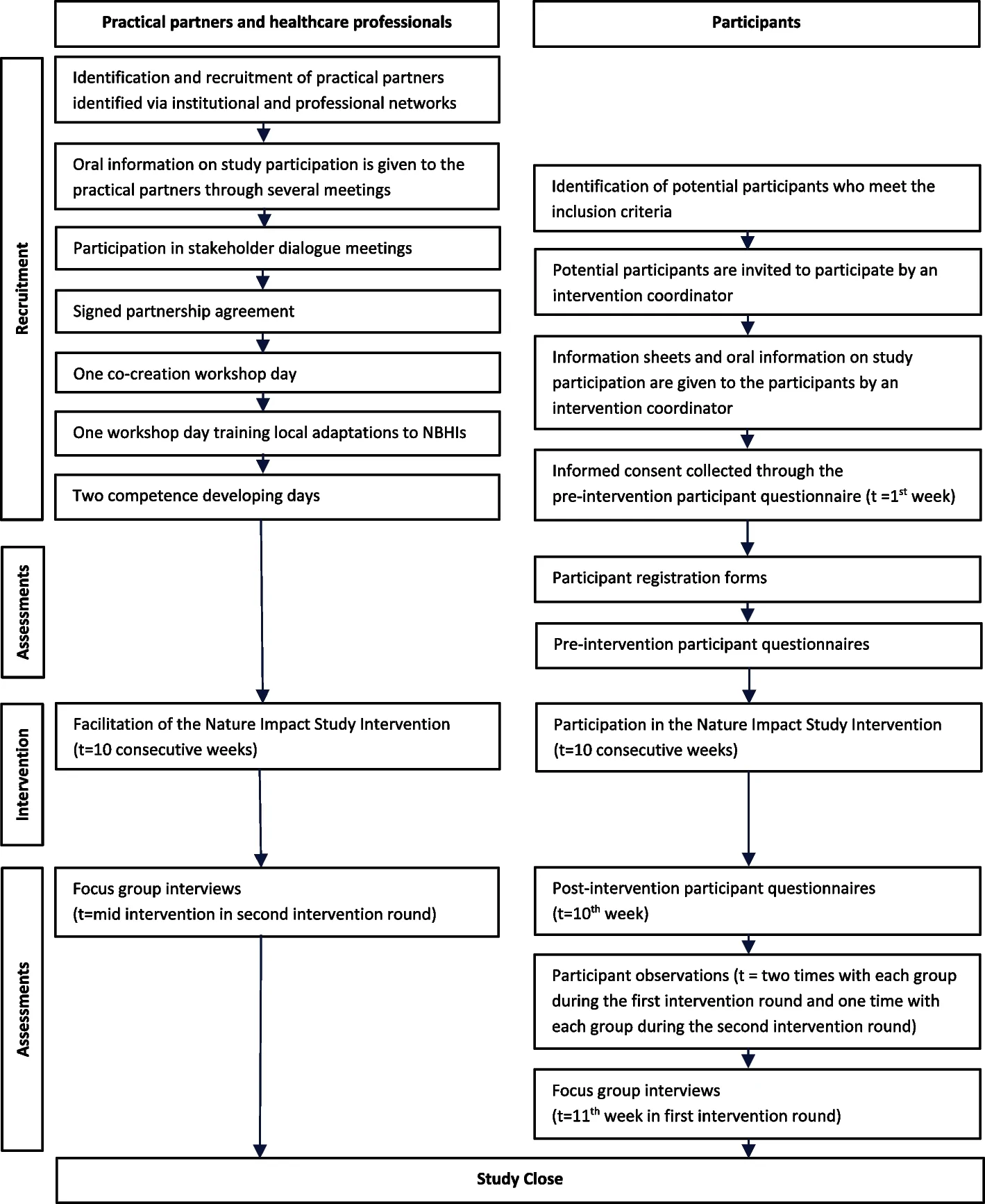
Time schedule of recruitment, intervention and assessments (SPIRIT) Legend: *t* indicates when the event occurs during the intervention. If unspecified, it applies to both rounds; if absent, the event occurs pre-intervention

The protocol has followed reporting guidelines for pilot and feasibility trials from Thabane and Lancaster [12] and follows the suggested utilisation of SPIRIT (Standard Protocol Items: Recommendations for Interventional Trials) [14], wherein some of the elements are replaced with elements from the CONSORT (The Consolidated Standards of Reporting Trials) [15] extension to pilot and feasibility trials. This combined approach is recommended because SPIRIT ensures completeness and structure, while the CONSORT extension tailors the focus to feasibility objectives such as recruitment, acceptability and trial procedures, which are more appropriate than definitive trial outcomes at this stage [12]. The adequately filled checklists for SPIRIT and CONSORT can be found in Additional files 1 and 2. Moreover, a SPIRIT figure, displaying a time schedule of enrolment, intervention and assessments, is displayed in Fig. 2.

### The structure of the intervention

The NBHIs are structured as group-based programmes delivered over 10 consecutive weeks, with one weekly session. The interventions will be delivered between March and November 2025. The intervention will be rolled out in two consecutive rounds, each involving between two and three groups at each site. Each group consists of 8 to 12 participants in a closed group. The closed group format, where the participants attend the same group for the full duration of the programme, is intended to provide participation continuity and to support the development of trusting relationships and psychological safety. Sessions held in the municipality centres have a duration of two and a half hours, while those at the outpatient clinic have a duration of one and a half hours. 

### Study setting

The intervention will be carried out as an interdisciplinary initiative facilitated by two health professionals at each site, who already have experience in delivering NBHIs to the target group. The Nature Impact Feasibility Study will be conducted at municipality centres in Kolding, in the Region of Southern Denmark, and Silkeborg, in the Central Denmark Region, and at an outpatient clinic in Glostrup, in the Capital Region of Denmark. The intervention conducted in Silkeborg will take place at two different natural environments. The sites are further described in Table 2. Across the three sites, contextual conditions are expected to vary, including differences in physical environments (e.g., urban versus more secluded natural settings), organisational structures and participant characteristics. These contextual variations are considered a central point of focus in the intervention, as they may influence both the implementation and the feasibility. To explore this, contextual factors will be systematically examined through qualitative and quantitative data sources, allowing for an analysis of how context shapes intervention delivery and feasibility outcomes across sites.

**Table 2:** Characteristics of the included sites

Location	Natural environment	Facilities
Silkeborg 1 Municipality	-Urban-adjacent forest with a lake -Bonfire area -Open lawn -Located near a folk high school -Publicly accessible and is used by the nearby folk high school -Light noise from highway	-Seat pads-Toilets
Silkeborg 2 Municipality	-Large hilly deciduous forest -Bonfire area -Open clearing -Publicly accessible and is used by school classes -No noise from traffic	- Seat pads -Warm water for drinks -Toilets
Kolding Municipality	-Hilly deciduous forest near a stream -Bonfire area -Located in a therapy garden -Only publicly accessible via agreements -No noise from traffic	-Seat pads -Snow suits -Warm water for drinks -Toilets
Glostrup Region	-Urban forest with a mix of deciduous and evergreen trees -Open clearing -Located near the outpatient clinic -Considerable noise from traffic and building sites	-Raincoats -Toilets in the nearby outpatient clinic

The research team will have continuous communication with the health professionals, to facilitate ongoing professional exchange and collaboration.

### Recruitment in the Nature Impact Feasibility Study

In the Nature Impact Feasibility Study, two distinct participant groups are recruited: (1) health professionals and (2) individuals experiencing mild to moderate anxiety, depression and/or stress, hereafter referred to as participants. The health professionals are working in the municipality centres in Kolding or Silkeborg or at the outpatient clinic in Glostrup.

Participants will first be approached through municipal centres, general practitioners, job centres, self-referral or the outpatient psychiatric clinic. Following this outreach, the health professionals delivering the interventions are responsible for identifying eligible participants, conducting screenings and delivering the intervention.

Inclusion criteria for participants include being 18 years or older, experiencing symptoms of mild to moderate anxiety, depression and/or stress that significantly affect daily functioning and having sufficient proficiency in Danish. Being on sick leave is not a requirement for participation. Exclusion criteria include having significantly reduced functional capacity, being unable to walk one kilometre, exhibiting suicidal ideation and/or experiencing substance abuse issues.

### Sample size justification in the Nature Impact Feasibility Study

As this study is designed to assess feasibility and implementation rather than intervention effectiveness, no formal statistical power calculation was performed [12, 15, 16]. The target sample size of 120 participants, distributed across three implementation sites, was determined to provide sufficient information on key feasibility and implementation outcomes, including reach, dose, fidelity, adaptations, acceptability, mechanisms of change and contextual factors influencing implementation. A sample of this size is expected to provide adequate variation in participant experiences and implementation processes across sites, enabling meaningful assessment of the feasibility of recruitment procedures, intervention delivery, data collection methods and implementation outcomes. The sample size was also considered appropriate to inform the design and planning of a subsequent larger-scale evaluation. This approach is consistent with recommendations that the design, including sample sizes, in feasibility studies should be justified in relation to feasibility objectives rather than statistical power for effectiveness testing [15, 16].

### Data sources

Six complementary data sources will be used to assess feasibility outcomes and to explore the appropriateness of outcome measures and the intervention’s potential to produce meaningful effects.

Data collection methods followed by data analysis procedures are summarised below, with each data source mapped to specific feasibility parameters following the CONSORT extension to pilot and feasibility studies [15]. The time schedule of enrolment and the assessments collected are displayed in Fig. 2.

#### Participant observations

Participant observations will be conducted during the interventions, with two observations per site in the first intervention round and one observation per site in the second intervention round. All observations cover a full session. The observations follow a participant observation approach inspired by James Spradley [17] and systematic field notes will be taken.

The observations will be carried out by a single trained researcher, who will participate in the sessions while maintaining a low degree of involvement. The use of systematic field notes, supported by an observation template, will ensure consistent attention to contextual elements such as group dynamics and therapeutic adaptations.

#### Focus group interviews

Semi-structured focus group interviews will be conducted with participants and health professionals separately. Separate interview guides will be developed for participants and health professionals, focusing on the overall feasibility objectives of the study, ensuring a consistent exploration of key aspects such as the implementation process, the participants’ and healthcare professionals’ experiences and contextual factors, while allowing for flexibility to raise unanticipated perspectives. The focus group interviews with the participants will be conducted after completion of the first intervention period whilst the focus group interviews with the health professionals will be conducted mid-intervention in the second round of interventions at each site. In total six focus group interviews will be conducted with the participants, and three focus group interviews will be conducted with the health professionals. The interviews are conducted by two researchers, a lead and a co-interviewer. All interviews will be audio recorded and transcribed verbatim.

Focus group interviews are selected due to their capacity to support with rich and diverse data created through social negotiations in group discussions [18]. The focus group interviews are particularly suited for examining feasibility parameters, as they allow for exchange and reflection among participants, which through the social interactions allow for broader perspectives, compared to individual interviews, enhancing understanding of the intervention’s acceptability and implementation process.

#### Participant registration forms

Prior to the start of the intervention, health professionals will complete a registration form for each referred participant, capturing information on participants’ entry/referral into the intervention and, if applicable, the reason for declining. The form includes open-text fields for the health professionals to fill out.

#### Participant questionnaires

Participants will be invited to complete two questionnaires assessing their mental health status and their relationship to nature using the validated instruments; WHO-5 Well-Being Index [19], Depression, Anxiety and Stress Scale (DASS-21) [20], Satisfaction with Life Scale (SWLS) [21] and Environmental Identity Scale [22]. These questionnaires are filled out pre- and post-intervention, right before the beginning of the first and at the end of the 10th session.

#### Intervention registration forms

Throughout the intervention, health professionals will complete a session log after each session. This log records any deviations from the planned intervention content, including changes in session timing and location, alongside the reasons for these adjustments, e.g., weather conditions or participant needs and preferences.

### Data analysis

#### Qualitative data analysis

Qualitative data from transcripts from the focus group interviews and the systematic field notes from participant observations will be analysed thematically following the approach described by Braun and Clarke [23, 24]. The analysis will be supported by NVivo software to facilitate coding and the identification of patterns across data sources.

The focus group interviews will be analysed to explore the feasibility parameters reach, adaptations, acceptability, mechanism of change and contextual factors alongside perspectives on secondary outcomes, related to the mental health status of participants and the assessment tools hereof. Moreover, the interviews with the health professionals will inform the feasibility parameter fidelity.

The systematic field notes from the participant observations as well as the open-text responses from the participant registration forms and the intervention registration forms will be thematically analysed [23, 24]. This analysis will contribute to an understanding of feasibility parameters including reach, fidelity, adaptation, acceptability, mechanisms of change and contextual factors.

#### Quantitative data analysis

Quantitative data will primarily be derived from participant questionnaires and structured elements of the participant and the intervention registration forms.

The participant questionnaires will be analysed using descriptive statistics to inform the feasibility parameters dose and reach as well as secondary outcome measures related to changes in participants’ mental health and their relationship to nature as well as the relevance of the chosen mental health outcome measures. Descriptive analyses will include measures of central tendency and distribution, e.g. means, medians, standard deviations, for each timepoint, alongside pre and post comparisons to examine a potential change.

Data from the participant registration forms will be analysed descriptively to assess referral sources and reasons for non-participation, thereby informing the feasibility parameter reach.

Data from the intervention registration forms will be analysed descriptively to assess information on the feasibility parameters, fidelity and adaptations as well as mechanisms of change.

All the data will inform contextual factors.

#### Data integration

A mixed methods convergent design will be applied, whereby qualitative and quantitative data are analysed separately and integrated during the interpretation phase. The integration will be guided by a thematic alignment. This approach allows for a comparative exploration of how qualitative insights and quantitative trends converge or diverge across feasibility parameters, mechanisms of change and contextual factors.

**Fig. 3:**
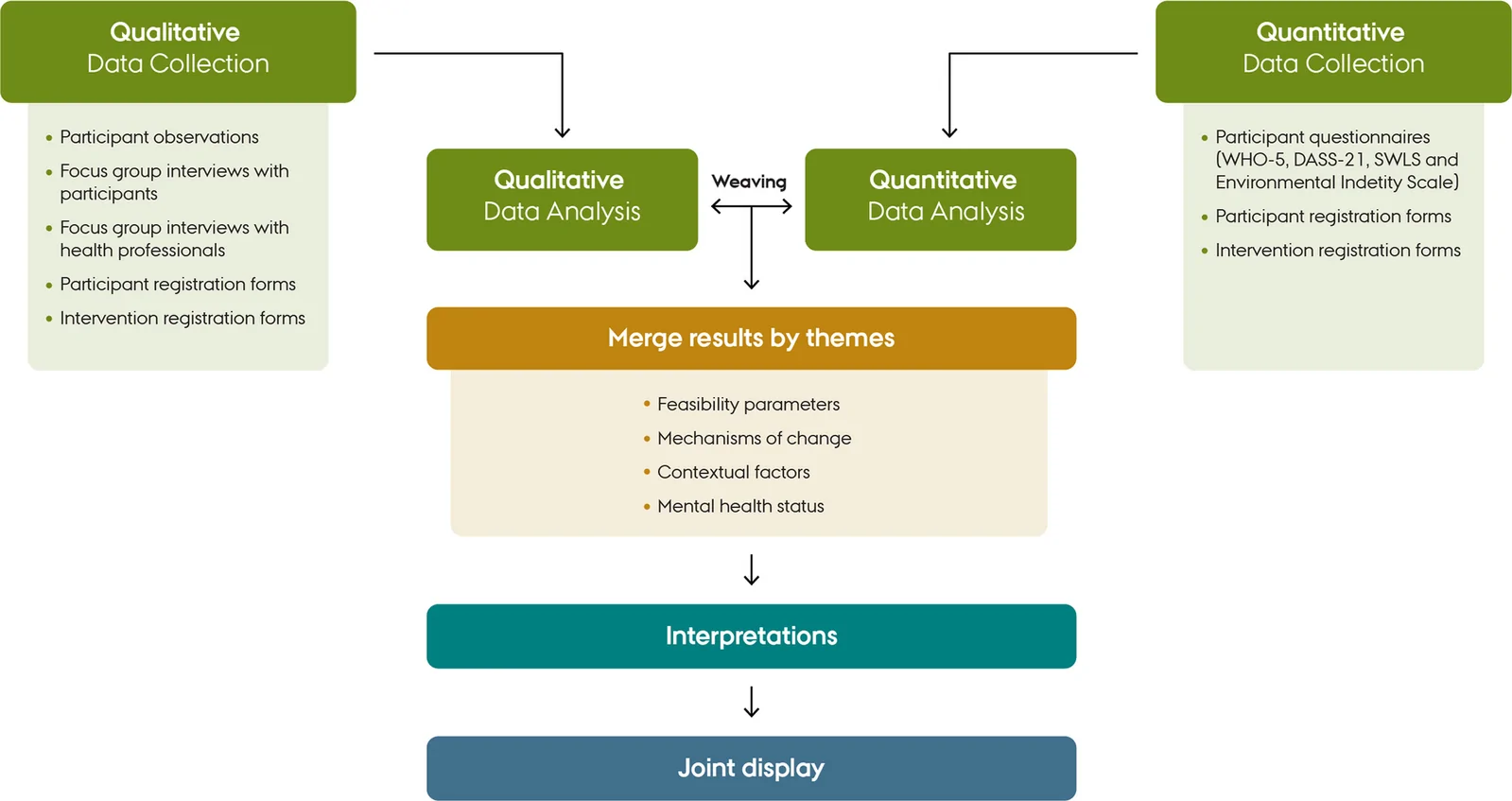
Overview of the planned mixed methods integration process

The two types of data will be merged in a weaving structure, which enables the presentation of complementary findings within thematic categories. Integration will involve systematically comparing and linking findings from qualitative and quantitative analyses within each domain to identify areas of convergence, divergence and complementarity. This approach is displayed in Fig. 3. A theme emerging from qualitative data can guide a focused exploration of a related pattern in quantitative data. Conversely, trends from quantitative data can prompt a deeper understanding through qualitative insights [13]. Where relevant, joint displays will be used to visually present integrated results, enhancing clarity and facilitating nuanced interpretation of feasibility outcomes.

To inform the decision on whether to proceed to a large-scale trial, predefined progression criteria have been established (Table 3). These criteria outline thresholds for key feasibility parameters and place them into three decision categories: Go, Amend and Stop. These thresholds were determined based on recommendations by Avery et al. [25]. The three decision categories indicate whether the study should proceed to a larger-scale trial, proceed with modifications, or not proceed without substantial changes.

**Table 3:** Progression criteria

	Go—proceed	Amend—proceed with changes	Stop—do not proceed unless changes are possible
Recruitment	≥ 90% of planned participants recruited	70–89% of planned participants recruited	< 70% of planned participants recruited
Adherence	≥ 90% of sessions completed with only minor, contextually justified adaptations	70–89% of sessions completed with only minor, contextually justified adaptations	< 70% of sessions completed with only minor, contextually justified adaptations
Participant retention	≥ 90% retention	70–89% retention	< 70% retention
Outcome measures	≥ 80% of outcome measures completed	70–79% of outcome measures completed	< 70% of outcome measures completed

*Legend*: Progression criteria align with the feasibility domains in Table 1: recruitment relates to reach; adherence relates to dose, fidelity, and adaptations; retention relates to acceptability; and outcome measures relates to the acceptability of measures relevant to the secondary aim

### Ethical considerations

All participants will receive both oral and written information about the purpose of the Nature Impact Feasibility Study, the intervention procedures and their rights as research participants. As part of the informed consent process, participants are informed about applicable data protection regulations (GDPR) and provided with contact information for the data controller.

Written informed consent is obtained from all participants prior to the initiation of any study procedures. After providing consent, participants are granted access to the participant questionnaires via a QR code, which directs them to a secure digital data collection platform. All interview recordings will be deleted following transcription, and the resulting transcripts will be pseudo-anonymised to prevent any statements from being linked to individual participants.

All data will be handled confidentially and will be stored on secure, access-restricted systems. Each participant is assigned a unique code that allows their responses to be linked across different time points (e.g. from baseline to post-intervention), without the possibility of re-identification. No key exists that could link these codes to personal identities. At the conclusion of the study, all data will be stored and, where appropriate, archived in accordance with institutional guidelines for data security and research integrity.

Moreover, the Nature Impact Feasibility Study is registered on ClinicalTrials.gov (NCT06981000) and has received exemption from Danish Research Ethics Committees (no. F-25008378).

## Discussion

The present study protocol reports the planned Nature Impact Feasibility Study, which is a mixed methods feasibility study designed to evaluate a contextually adapted, group-based NBHI for adults experiencing mild to moderate symptoms of anxiety, depression and/or stress. The protocol describes the systematic development of the intervention, the programme theory and logic model guiding the intervention and the feasibility evaluation across the three core domains: implementation, mechanisms of change and contextual factors.

### Expected contributions

The Nature Impact Feasibility Study will evaluate the feasibility of a contextually adapted NBHI for individuals experiencing mild to moderate symptoms of anxiety, depression and/or stress. This is done through an investigation of the three core domains. Moreover, the study assesses the suitability of the chosen outcome measures and offers insight into a potential tendency of an effect.

As the first Danish feasibility study of its kind, it contributes valuable knowledge on how such interventions can be designed, delivered and studied within both municipal and regional contexts.

The Nature Impact Feasibility Study provides a foundation for the future development and evaluation of NBHIs. It will present a practice-oriented programme theory and logic model that offers a structured yet flexible framework for professionals working with individuals experiencing anxiety, depression and/or stress. This framework will be designed to be adaptable across different settings, making it relevant for use in diverse geographical and contextual environments where contextual factors may vary.

### Strengths and limitations

The Nature Impact Study’s mixed methods design enables an in-depth feasibility evaluation. By combining qualitative and quantitative data from multiple sources, the study contributes to understanding key implementation parameters. However, the mixed methods design can be demanding. Maintaining coherence and clarity may be challenging, particularly when qualitative and quantitative findings are reported in thematic alignment.

Besides the demanding complexity of the mixed methods design other limitations should be noted. The participating sites, which before the initiation of the Nature Impact Feasibility Study were involved in mental healthcare, may be more inclined toward health-promoting initiatives than others. Additionally, participant observations are conducted by a single researcher, raising the risk of bias, although this was addressed through team debriefing and reflexivity.

### Dissemination

Based on the findings from the Nature Impact Feasibility Study, several scientific articles and an intervention manual will be developed. These outputs are intended for publication in various academic journals, including but not limited to the categories: (1) environmental and nature-based health, (2) mental health and clinical psychology and (3) health, lifestyle and physical activity. The articles and the manual are expected to be published during 2025 and 2026.

In summary, this study protocol promotes transparency for the upcoming Nature Impact Feasibility Study, enabling critical evaluation of its proposed design and methods. Together with the subsequent Nature Impact Feasibility Study, this will provide a strong foundation for future research on NBHIs, via the complex exploration of the feasibility parameters through a mixed methods design. Furthermore, the logic model provides a practical and adaptable tool for use across a wide range of mental health promotion efforts. The study contributes essential knowledge that can inform future large-scale evaluation and effect studies and the practical application of NBHIs for individuals experiencing mild to moderate anxiety, depression and/or stress.

## Supplementary Information


Additional file 1.Additional file 2.

## Data Availability

The manuscript has no associated data.

## Abbreviation

NBHI: Nature-based health intervention
CONSORT: The Consolidated Standards of Reporting SPIRIT: Standard Protocol Items: Recommendations for Intervention Trials
MRC: Medical Research Council
WHO-5: World Health Organization-Five Well-Being Index
DASS-21: Depression, Anxiety and Stress Scale SWLS: Satisfaction with Life Scale
